# Estimation of Tooth Dimensions and Golden Divine Ratio in Extracted Human Permanent Maxillary and Mandibular Canines in a Cohort of Tamil Ethnicity

**DOI:** 10.7759/cureus.54854

**Published:** 2024-02-25

**Authors:** Sathya M Kumar, Deepak Pandiar, Reshma Poothakulath Krishnan, Ramya Ramadoss

**Affiliations:** 1 Oral Pathology and Microbiology, Saveetha Dental College and Hospitals, Saveetha Institute of Medical and Technical Sciences, Saveetha University, Chennai, IND; 2 Oral Pathology and Oral Biology, Saveetha Dental College and Hospitals, Saveetha Institute of Medical and Technical Sciences, Saveetha University, Chennai, IND

**Keywords:** golden ratio, root length, permanent teeth, indian, crown length, canines

## Abstract

Background

Teeth serve many functions, and aesthetics is one of the most important aspects served by teeth, perceived by the limbic system of the human brain. The golden divine ratio is the unique proportion often correlated with beauty. The present study was devised to estimate the dimension of human permanent canines and approximation to the golden divine ratio.

Materials and methods

The present study included 47 extracted human permanent canines retrieved from the tooth repository of our institute's Department of Oral Biology. Using digital vernier calipers (Themisto TH-M61 digital vernier caliper, 0-150mm/ 6 inch, JIPVI Ecommerce Pvt. Ltd, India, 2022), the following measurements were taken: Mesio-distal and labiolingual dimensions of the crown, crown length, root length, root to crown ratio (R/C) and the tooth to root ratio (T/R). The data were analyzed using Statistical Package for Social Sciences (SPSS) software version 26.

Results

All the dimensions' mean and standard deviations were calculated for both maxillary and mandibular canines. While the means of mesio-distal and labiolingual dimensions of the crown approximated the values reported in the literature, there was some variation in crown and root lengths. The mean crown lengths of the upper and lower canines were 10.34mm and 9.76mm, respectively, while the root lengths were 16.52 and 15.54mm, respectively. The R/C of both sets and the T/R of the upper canine only followed the golden ratio. T/R of the lower canine was slightly higher (1.64)

Conclusion

Although the number of included teeth was less, owing to the fact that canines are rarely extracted, our results provided new values of canines for updation in a unique population. More studies are required for comparative anthropological data updates.

## Introduction

The golden divine ratio (GDR) is a unique proportion, and it has been hypothesized most 'aesthetically pleasing objects or subjects' bear this ratio, set at 1.618 (Phi/ɸ) [1]. Human teeth comprise 60% of our oral cavity and are functionally important and significant for aesthetics. The multifaceted relevance of teeth, from providing attractive aesthetic smiles to the function of load bearing and mastication, methodical analyses have demonstrated that these principles can be employed for evaluation and altering dental aesthetics with predictability and thus may be considered, identified, evaluated, and developed independently in aesthetic management. One of the aesthetic principles is the proportion, which can be predicted by a formula deriving a ratio of one component to another.

A golden ratio of 1.618 or a golden proportion of 62% is usually considered aesthetically pleasing when it concerns the teeth [2]. While most researchers find it fascinating, available data contracts the concept of correlation between aesthetics and the golden ratio. Preston's preliminary study on 58 dental casts found that upper anterior teeth do not follow GDR and considered this ratio 'unrealistic' [3]. Brisman, instead of divine ratio, used a different approach [4]. Recently, Agou et al. estimated that a width proportion for lateral to central incisors is 77% instead of a golden proportion of 62% and considered 77% as the most appropriate proportion for 'the most attractive smiles', which also contrasts the concept of golden divine ratio [5].

Another important consideration regarding teeth dimensions stems from the fact that the teeth measurements we follow were originally published in the 1940s, and there has been no update in the values since then [6]. Teeth are among the few human body parts that not only show sexual dimorphism but also bear great diversity in shapes, sizes, and structure based on geographic distribution, ethnicity, and race. There is a need to analyze and present updated teeth dimensions from different geographic locations for anthropological updation. The present study was devised to analyze the dimensions of human permanent maxillary and mandibular canines and approximation to the golden divine ratio.

## Materials and methods

Study sample and preparation of teeth

The present study included 47 extracted permanent human extracted permanent canines retrieved from the institutional tooth repository, SDC Vivarium. The study was conducted in the Department of Oral Pathology and Microbiology in collaboration with Oral Biology in a tertiary health care center in South India after the ethical approval was granted by Saveetha Dental College-Institutional Human Ethical Committee (SDC-IHEC) with approval number IHEC/SDC/UG-2055/23/OPATH/018. One hundred twenty-seven canines were examined initially for inclusion in the study. After excluding eighty attritted, fractured, carious, and severely deformed canines, 47 sound teeth were included for further evaluation. The teeth were manually scaled for removal of any debris and soaked in sodium hypochlorite solution. Out of 47 canines, there were fifteen permanent maxillary canines and 32 extracted human permanent mandibular canines.

Estimation of teeth dimensions

**Figure 1:**
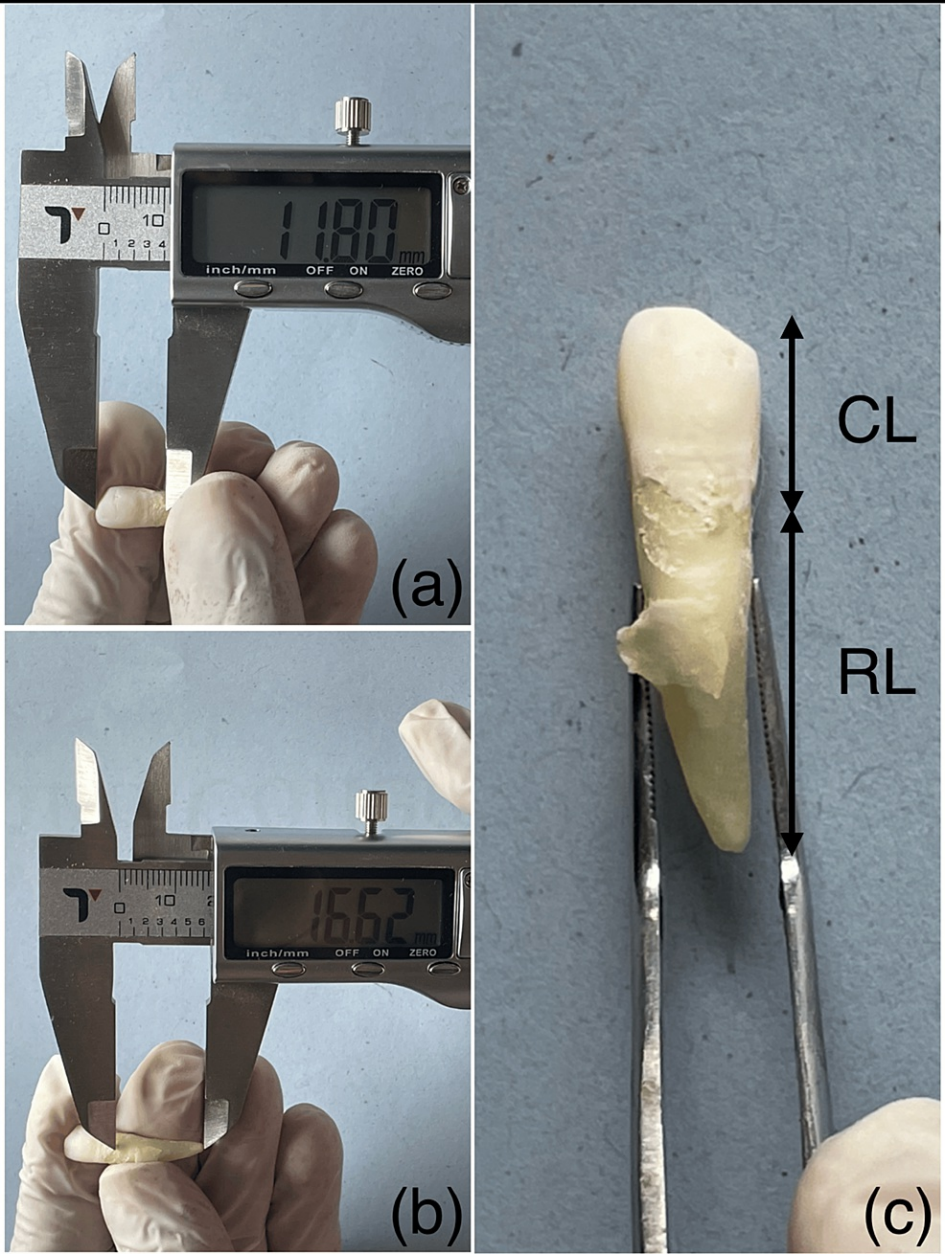
a) Photograph showing estimation of crown length from incisal edge to cemento-enamel junction (CEJ), b) Photograph showing estimation of root length from CEJ to root apex, and c) Diagram depicting estimation of measurement for Golden ratio derivation.

To measure the mesio-distal width, labiolingual dimensions, and the lengths of the roots and crown, we employed a set of digital vernier calipers with a minimum count of 0.01 inch/0.02 mm (Themisto TH-M61 digital vernier caliper, 0-150 mm/ 6 inch, JIPVI Ecommerce Pvt. Ltd, India, 2022). For measuring mesiodistal and labiolingual dimensions, the measurements were taken at the maximum width; to estimate the length of the tooth crown, the facial aspects were measured from the tips to the deepest portion of the cementoenamel junction (CEJ). Likewise, the length of the roots was estimated from the deepest point in the CEJ to the apex (Figures 1a-1c).

Estimation of Golden divine ratio

GDR was estimated as previously described [1]. Using digital vernier calipers, we first estimated the length of the crowns and roots separately and recorded them. After that, two ratios were calculated individually for all the included teeth: a) the ratio of the length of root (RL) to the length of the crown (C), recorded as RL/CL, and b) the ratio of total tooth length (TL) to root-length (RL), recorded as TL/RL. These ratios were correlated as follows for determining GDR: RL+CL:RL=RL: CL= φ (1.618).

Statistical analysis

The measurements of mesiodistal and labiolingual dimensions of the crowns and root and crown lengths of upper and lower permanent canines were determined and entered into the Microsoft Excel spreadsheet in 2021. The means and standard deviation of mesiodistal width, labiolingual width, length of the crown, root length, total tooth length, TL: RL, and RL: CL were analyzed using SPSS software version 26 (Released 2019; IBM Corp., Armonk, New York, United States).

## Results

Maxillary permanent canine

The average mesiodistal width (MDW) for permanent maxillary canine was 7.3±0.53 mm, while the labiolingual dimension (LLW) was 7.7±0.09 mm. The crown length, root length, and total length were 10.34± 1.01 mm, 16.52±0.66 mm, and 26.75±1.66 mm, respectively. Fascinatingly, RL/CL and TL/RL ratios were within the golden divine ratio. The RL/CL ratio for maxillary canine was 1.61±0.16, and the total length to root length (TL/RL) ratio was 1.62±0.093.

Mandibular permanent canine

Similar values were approximated for lower canines. The MDW and LLW were 7.2mm and 7.4mm, respectively. About CL, RL, and TL, the values were 9.76±0.93, 15.54±1.68 and 25.46±1.87 mm, respectively. The GDR calculated for root length: crown length was 1.607±0.25 and 1.64±0.087 for TL/RL (Table 1).

**Table 1:** Mean mesiodistal width, labiolingual width, crown length, root length, total length, mean R/C ratio and mean tooth-root ratio for maxillary and mandibular canines MDW- mesiodistal width, LLW- labiolingual width, SD- standard deviation, CL- crown length, RL- root length, TL- total length, R/C- root to crown ratio, T/R- total length to root length ratio L- root length, TL- total length, R/C- root to crown ratio, T/R- total length to root length ratio

Tooth Type (n=number)	MDW (in mm ±SD)	LLW (in mm ±SD)	CL (in mm ±SD)	RL (in mm ±SD)	TL (in mm ±SD)	R/C	T/R
Maxillary canine (n=15)	7.3±0.53	7.7±0.09	10.34±1.01	16.52±0.66	26.75±1.66	1.61±0.16	1.62±0.093
Mandibular canine (n=32)	7.2±0.14	7.4±0.32	9.76±0.93	15.54±1.68	25.46±1.87	1.607±0.25	1.64±0.087

## Discussion

Canines, usually regarded as the corner teeth of human dentition, are unique in location and function. In dentistry, canines' aesthetic and functional values are very helpful in providing many major purposes, such as designing, guidance, and retention. Thus, any sort of dental treatment provided to the patient should aim to restore natural and aesthetic appearance and function [7]. In devising a treatment strategy for missing anterior teeth, dentists should attempt to replicate both individual variations and optimal anatomic and facial aesthetic parameters. In the present study, we examined 47 extracted permanent human teeth; a smaller sample size is attributed to two facts: firstly, sound canines are rarely extracted unless deemed necessary, and secondly, a large number of teeth were excluded, even if there were minimal defects to avoid inappropriate readings. Most of the values and dimensions followed to date are decades old and have not been updated [6]. Providing data about the dimensions of teeth is not only necessary for treatment but also for comparative anthropology and forensic odontology.

Canines, usually regarded as the corner teeth of human dentition, are unique in location and function and show great dimorphism [7]. In dentistry, canines' aesthetic and functional values are very helpful in providing many major purposes, such as designing, guidance, and retention [8]. Thus, any sort of dental treatment provided to the patient should aim to restore the natural and aesthetic appearance and function [9]. In devising a treatment strategy for missing anterior teeth, dentists should attempt to replicate both individual variations and optimal anatomic and facial aesthetic parameters. Previous studies have analyzed oral hard and soft tissue morphometrically [10]. In the present study, we examined 47 extracted permanent human teeth; a smaller sample size is attributed to two facts: firstly, sound canines are rarely extracted unless deemed necessary, and secondly, a large number of teeth were excluded, even if there were minimal defects to avoid inappropriate readings. Most of the values and dimensions followed till date are decades old and have not been updated [6]. Providing data about the dimensions of teeth is not only necessary for treatment but also for comparative anthropology and forensic odontology.

In the present study, we aimed to measure canines in their linear dimensions and estimate the approximation to the golden ratio. Most of the previous studies have estimated the MDW, LLW, and CL in human participants or models/casts, with minimal data on the golden ratios or the length of the roots [11-14]. Conventionally, the MDW of upper and lower canines are set as 7.5 and 7.0mm, respectively, and 8.0 and 7.5mm are the respective LLW for maxillary and mandibular canines [6]. In contrast, in a subset of the Tamil cohort, we found that these dimensions were lesser in both sets of teeth by an mm or two. Alanazi AA et al., in their study of the Saudi Arabian population, compared the MDW, LLW, and CL in maxillary and mandibular canines in both genders and opined that the morphometric analysis of permanent canines is fruitful in gender determination [11]. Similar to our study, their values were lower than standard values, and females had even smaller canines [11]. The authors found a significant dimension difference among the canines from all four quadrants. Another study from South India found that the maxillary canines showed a mean of 7.73 mm, which was even larger than the standard dimensions [9]. The mandibular canines were similar in dimensions, depicting that maxillary canines show more morphometric variation globally [15-16].

For aesthetic rehabilitation and restoration, various tooth proportions/ratios have been tested in different populations; while most researchers prefer the establishment of a golden ratio of 1.618 or a proportion of 62%, contrasting opinions have been put forward by other authors [3-5]. While the golden ratios of the TL/RL or RL/CL and the width/height ratio of human canines could be useful guides in reestablishing an attractive smile and for functional purposes, they may also be used in forensic medicine for identification and comparative anthropological studies. Anand et al. found that from Western India, RL/CL and TL/RL for maxillary canines (10 teeth) were 1.636±0.015 and 1.605±0.007, respectively [1]. In our cohort of Tamil ethnicity, the values approximated 1.61±0.16 and 1.62±0.093. Thus, despite being smaller in crown lengths, maxillary canines in our population approximate GDR. Regarding the mandibular canines, we had a larger number of teeth. The GDR calculated for RL/CL was 1.607±0.25 and 1.64±0.087 for TL/RL; the reverse ratio was noted in another study comprising 10 mandibular canines (1.633±0.014 for RL/CL and 1.606±0.005 for TL/RL) [1].

Our results indicate clinicians must establish a naturally accepted tooth proportion, a more desirable crown width-to-length ratio, and side symmetry when restoring or replacing maxillary anterior teeth. Further studies should investigate other methods of determining and differentiating the individual-functional form of anterior canine teeth. A larger sample size may be needed before extrapolating these results to the general population.

Limitations

As the maxillary canines are rarely extracted, the number of extracted maxillary canines was comparatively less. Further, the cases were not segregated based on gender, which would have further diluted the number of included teeth. We used vernier calipers for morphometric analysis. Thus, the curved morphologies could be underestimated in a few cases, for which brass wire may be used in future studies.

## Conclusions

Within the limitations of the present study, we hereby report the dimensions of permanent canines. The canines in our South Indian population are comparatively smaller but still follow the golden divine ratio. These baseline values may be used while planning the placement of dental implants, wherein implant lengths should correspond to the ratio of phi.
